# Supplementary material for: Waveguide coupled III-V photodiodes monolithically integrated on Si
Source: Nat Commun. 2022 Feb 17;13:909. doi: 10.1038/s41467-022-28502-6 (PMC8854727; doi:10.1038/s41467-022-28502-6)
Supplement: Supplementary file 1 — Supplementary Information [file 41467_2022_28502_MOESM1_ESM.pdf]

Supplementary Information

Waveguide coupled III-V photodiodes monolithically integrated on Si

Pengyan Wen[1], Preksha Tiwari[1], Svenja Mauthe[1], Heinz Schmid[1], Marilyne Sousa[1], Markus Scherrer[1], Michael Baumann[2], Bertold Ian Bitachon[2], Juerg Leuthold[2], Bernd Gotsmann[1] and Kirsten E. Moselund[1,✉]

[1]*IBM Research Europe – Zurich, Säumerstrasse 4, 8803 Rüschlikon, Switzerland*

[2]*ETH Zürich, Institute of Electromagnetic Fields (IEF), Gloriastrasse 35, 8092 Zürich, Switzerland*

✉ email: kirsten.moselund@epfl.ch

Supplementary Note 1: Characterization setups

1.1 Scanning thermal microscopy (SThM). In this work, we use scanning thermal microscopy (SThM) for the characterization of thermal effects of the device. Supplementary Figure 1 shows the schematical overview of the SThM thermometry technique. The method and setup have been described before [1]. Briefly, this SThM-based technique relies on a micro-cantilever with integrated resistive sensor coupled to the silicon tip. The temperature of the tip T_{sensor} (out of contact) = 267 °C is known by detecting the lever voltage and we calibrate it before the scan when the tip is out of contact. The measurement is performed in high vacuum (<10^{-6} mbar) at room temperature in the Noisefree labs at IBM Research Europe - Zurich [2]. The scan is operated in contact mode of scanning force microscopy. The contact force is monitored and controlled with a laser deflection system. The temperature of the sample is modulated by applying an AC voltage with frequency of f_{mod} = 1 kHz on the device and a series resistance R_s = 10 kΩ. We apply a voltage including AC and DC components to distinguish the influence of current direction (forward and reverse) on the self-heating, $V_{applied}$

= $V_{applied\_DC}$ + $V_{applied\_AC}$ sin $(2\pi f t)$. The results shown in the main text (Fig. 3) are carried out applying $V_{applied\_DC}$ = 0 and $V_{applied\_AC}$ = 4 V. This induces an AC voltage on the device between −3.8 V and +3 V. Therefore, the measurement contains information on forward and reverse direction. To test the reverse direction separately, measurements are also done applying $V_{applied\_DC}$ = −4 V and $V_{applied\_AC}$ = 0.2 V, as shown in Supplementary Figure 4.

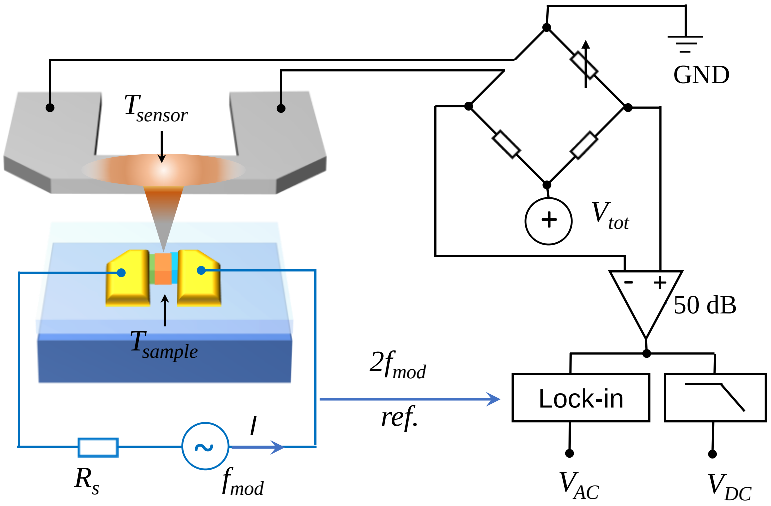

Supplementary Figure 1 Schematical overview of the SThM thermometry technique applied in this work. An AC bias with frequency f_{mod} is applied on the T-shape p-i-n photodiode with a series resistance R_s. The resulting AC current with amplitude I leads to a periodic Joule self-heating with frequency $2f_{mod}$. The temperature of a local spot on the sample surface is thermally coupled through the tip to a resistive sensor integrated in the cantilever. The changes of the sensor temperature T_{sensor} lead to changes of its electrical resistance, which are tracked using a Wheatstone bridge circuit. The DC change in voltage V_{DC} between the legs of the Wheatstone bridge and its AC amplitude V_{AC} at $2f_{mod}$ relate the local sample temperature T_{sample} to the kown and constant out-of contact sensor temperature through equation (1).

The local spot on the sample with temperature T_{sample} is thermally coupled through the tip to a resistive sensor integrated in the silicon MEMS cantilever. The change of the sensor

temperature T_{sensor} leads to the change of the electrical resistance of the cantilever, which is tracked using a Wheatstone bridge circuit. The DC change in the measured voltage ΔV_{DC} between the legs of the Wheatstone bridge and its AC amplitude ΔV_{AC} at $2f_{mod}$ relate the local sample temperature T_{sample} to the known out-of-contact sensor temperature through the following equation:

$$\Delta T_{sample} = \Delta T_{sensor} \text{ (out of contact)} \times \frac{\Delta V_{AC}}{\beta \Delta V_{DC} - \Delta V_{AC}} \qquad (1)$$

where $\beta \neq 1$ can correct for an applied voltage with $V_{applied\_DC} \neq 0$.

1.2 Free-space electrical and photoluminescence setup. This setup is used for the electrically pumped emission measurements as well as for evaluation of the spectral response of detection, both are carried out using free-space coupling in reflection mode. The layout of the setup is schematically shown in Supplementary Figure 2. Red illustrates the light path for detection, whereas yellow indicates the light path for excitation. For the optical pumping we use a ps-pulsed laser which allows to adjust the excitation wavelength from about 640 nm to 2000 nm with a fixed repetition rate of 78 MHz (NKT SuperK supercontinuum EXR-15 source with NKT SuperK SELECT+ broadband tunable wavelength filter). The excitation source has a gaussian shape with a spot size of around 1 µm. In all experiments the sample is placed in a custom-made optical cryostat from CryoVac where light is focused on the device using an objective with a magnification of 100× and a numerical aperture of 0.6 placed inside the cryostat. The light is collected by the same objective and detected by an InGaAs line array detector (Princeton Instruments, PyLoN-IR 1.7) which is combined with a grid diffraction spectrometer (Princeton SP-2500i). Four electrical probes are placed within the cryostat, which allows to either apply a reverse bias in the detection mode or a forward bias to observe the electroluminescence.

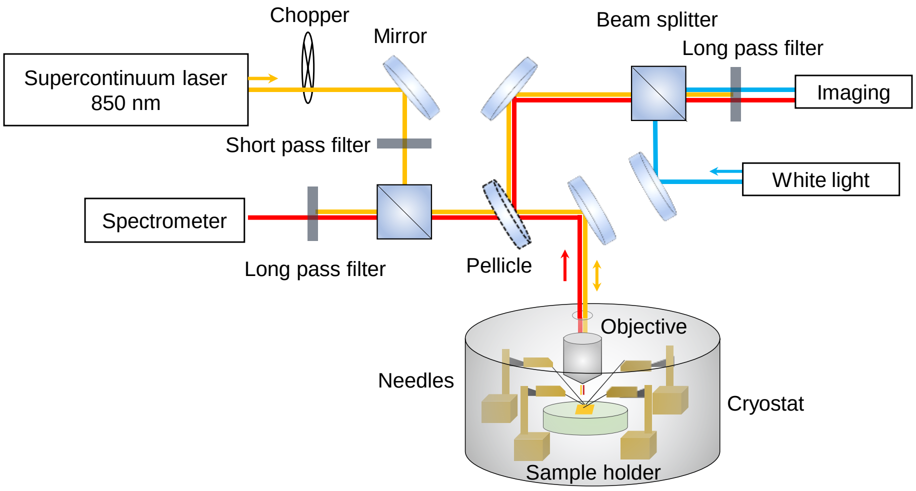

Supplementary Figure 2 Schematical overview of the electrical luminescence and power dependent detection measurement setup. The red line indicates the light path for detection, whereas the yellow line indicates the light path for excitation. The main parts include a supercontinuum laser (excitation wavelength from about 640 nm to 2000 nm with a fixed repetition rate of 78 MHz), a custom-made optical cryostat (inside there are four needles, an objective with a magnification of 100× and a numerical aperture of 0.6), a grid diffraction spectrometer with an InGaAs line array detector and an imaging system with white light source.

1.3 Bandwidth measurement and data transmission setup. Supplementary Figure 3 shows the bandwidth and data transmission setup. For the data transmission experiments, an electrical data signal is first generated using a Mircram 100 GSa/s digital to analog converter (DAC). The output of the DAC is then amplified using a SHF driver amplifier (DA) with 3dB bandwidth of 55 GHz. A u2t Mach-Zehnder modulator is then used to transfer the electrical signal to the 1320 nm optical carrier generated with a Keysight tunable laser source (TLS). We then amplified the optical signal to the optimum power using a FiberLabs Praseodymium doped fiber amplifer (PDFA). High-speed RF probes are used to extract the RF signal from the device under test (DUT) and a reverse bias voltage of −1.5 V is supplied to the DUT using a SHF bias tee. At the receiver, an Agilent 160 GSa/s digital storage oscilloscope (DSO) is used to record

the generated RF signal. A 30 cm RF cable is used to connect the DSO to the bias tee, limiting the frequency response of the full system. We used an offline digital signal processing to process the recorded signal. The digital signal processing step comprises of signal normalization, timing recovery, linear equalization, and non-linear equalization.

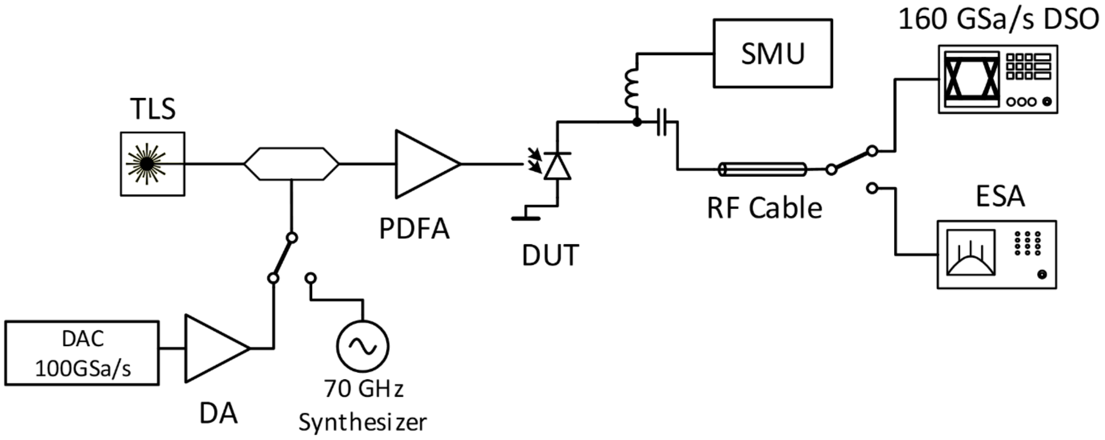

Supplementary Figure 3 Schematical overview of the high-speed setup for bandwidth and data transmission measurements. The signal generation part includes a digital to analog converter (DAC) and a driver amplifier (DA). The electrical signal is then transferred to optical carrier generated with a Keysight tunable laser source (TLS). The optical signal is amplified with the optimum power using a FiberLabs Praseodymium doped fiber amplifer (PDFA). High-speed RF probes are used to extract the RF signal from the device under test (DUT). An Agilent digital storage oscilloscope (DSO) is used to record the generated RF signal at the receiver. A 30 cm RF cable is used to connect the DSO to the bias tee, limiting the frequency response of the full system.

For the bandwidth measurements, a continuous wave (CW) tone is generated using a 70 GHz Keysight Synthesizer. This tone is modulated onto a 1320 nm optical carrier. The detected tone is measured using a 110 GHz Keysight spectrum analyzer (ESA). The system frequency response is calibrated using a commercial 67 GHz u2t photodiode. A calibration frequency

response is measured using the commercial photodetector (PD). Using datasheet values for the PD response, the response of the modulator and cables could be calibrated. For the RF probe, datasheet values are taken as well to have a full system frequency response calibration.

Supplementary Note 2: Material growth

MOCVD growth. The MOCVD growth is performed on a 2×2 cm chip. The patterned chip is cleaned by performing a wet clean in a heated piranha solution (70 °C), which ensures a clean SiO_2 template and prevents parasitic nucleation. Then a 20 s DHF dip is performed to remove the native oxide on the Si seed in the empty SiO_2 template.

Supplementary Table 1 MOCVD growth information.

Material	Growth duration [s]	TMIn flow [µmol/s]	TMGa flow [µmol/s]	TBA flow [µmol/s]	TBP flow [µmol/s]	DEZn flow [µmol/s]	TmSn flow [µmol/s]
InAs	50	0.028	-	6.5	-	-	-
n-InP	180	0.110	-	-	10.5	-	0.0017
i-InGaAs	360	0.033	0.127	4.95	-	-	-
p-InP	420	0.028	-	-	7.45	0.092	-
p-InGaAs	300	0.033	0.127	4.95	-	0.074	-

Supplementary Table 1 depicts the different molecular flow ratios during MOCVD growth. All growth steps are performed at 550 °C. First, a short InAs (50 s) which selectively nucleates at the Si interface is grown to improve nucleation compared to directly growing InP. Next, n-InP is grown using TMIn, TBP precursors with Sn doping from TMSn. Then, the i-InGaAs is grown using TMIn, TMGa and TBA. In the next step, the p-InP is grown by using TMIn and TBP presursors with Zn doping from DEZn. At last, the p-InGaAs is grown by using TMIn, TMGa and TBA presursors with Zn doping from DEZn. At the end of the growth, the temperature is ramped down. Due to the growth in an oxide template, no post processing is required. However, if the crystal grew out of the template, it can be removed by performing a

polishing process. The width and thickness of the III-V segments are determined by the geometry of the pre-patterned Si layer while the length is controlled by the growth duration.

Supplementary Note 3: Characterization

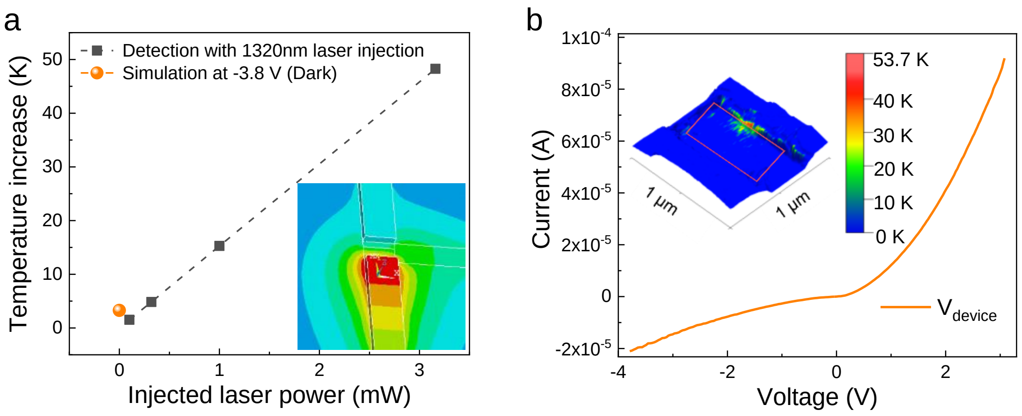

Supplementary Figure 4 Thermal effects of p-i-n device operating as detector. a, Simulated temperature dependence on laser power injection. The inset shows the simulated temperature distribution at -3.8 V without light injection. **b**, Current-voltage diagram of the device. The inset shows SThM result showing topography and color-coded temperature rise of the device working as detector with applied voltage of –3.8 V on the device (Dark).

3.1 Thermal effects while operating as detector. Supplementary Figure 4 shows the device thermal effects while operating as detector. The black squares in Supplementary Figure 4a show the temperature dependence on the injected laser power. In this simulation, we assume a Gaussian distributed laser spot centered at 1320 nm directly injected at the i-InGaAs region. Considering the photocurrent generated in the main text (Fig. 5), we could assume that all the optical power transferred into heat. The temperature increase under an injection power of 3.16 mW is around 50 K, which is within the acceptable range during operation. The orange disk shows the simulation result at –3.8 V without laser injection (Dark) with a temperature increase of 3.3 K. Supplementary Figure 4b shows the I-V curve of the device with inset a SThM result measured under V_{device} = –3.8 V without laser injection, we hardly see any temperature increase

of the major III-V region (red rectangle) except for a local high temperature region at the contact edge. This local high temperature could be due to the local high resistance between III-V and metal.

3.2 Electrical/electro-optical luminescence measurements. Supplementary Figure 5a shows the EL spectra plot in photon energy and Supplementary Figure 5b shows the EL peak energy dependence on current at various temperatures. All the EL spectra are measured under the application of DC forward bias, applied using an electrical parameter analyzer B1500 from Agilent.

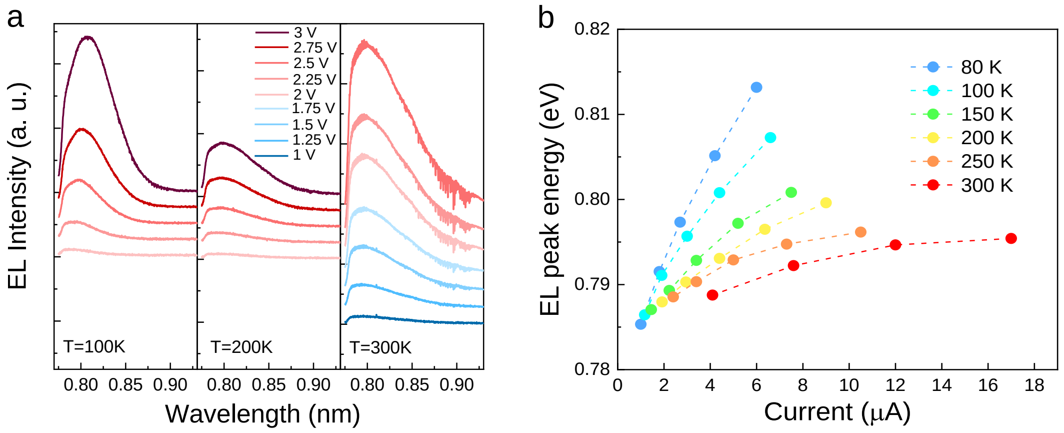

Supplementary Figure 5 Temperature dependent EL spectra plotted in photon energy. a, Electroluminescence (EL) spectra of a 350 nm wide T-shape device (T3) under continuous wave (CW) forward bias measured at 100 K, 200 K and 300 K. **b**, EL peak energy dependence on injection current at temperature from 80 K to 300 K.

3.3 Waveguide propagation and coupling losses. To assess the loss in the waveguide, we measured the transmission of waveguides with various lengths from 300 μm to 10 mm with a grating coupler at either end, which reveals a transmission loss of 5 dB/mm. All the waveguides are with width of 500 nm. The result is shown in Supplementary Figure 6a. We also measured several waveguides with length of 300 μm (shown in Supplementary Figure 6b) and they show uniform transmission loss of 12 dB at 1320 nm. This translates to a 6 dB per coupler in the

devices we demonstrated, and we use this value to calibrate the responsivity data in the main text (Fig. 5). As also mentioned in the paper, additional coupling losses from the silicon waveguide to the active III-V region are not accounted for.

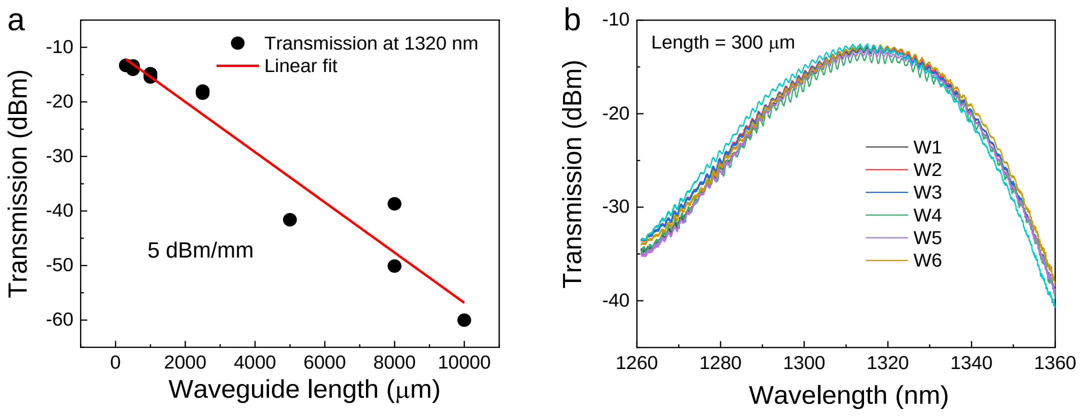

Supplementary Figure 6 The silicon waveguide transmission. a, Wavelength dependent transmission of silicon waveguide with various lengths at 1320 nm and linear fit determining transmission losses of around 5 dB/mm. **b**, Wavelength dependent transmission of the 300 μm waveguides showing uniform transmission loss of 12 dB at 1320 nm.

Supplementary Figure 7a shows the free-space spectral dependence of I-V curves with wavelengths from 1200 nm to 1600 nm, which show a reduction of detection current with the increase of wavelength. In Supplementary Figure 7b, we compare the behavior with the absorption coefficient of 53% InGaAs taken from literature [3]. The spectral dependence of the p-i-n device complies well with the expected response InGaAs 53% between 1200 nm and 1600 nm.

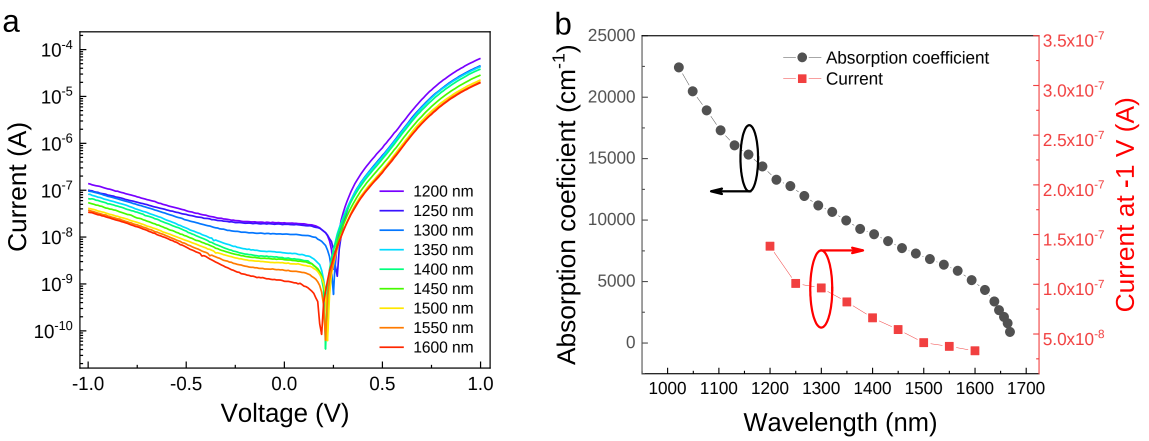

Supplementary Figure 7 Static electrical characterization of detectors. a, Free-space spectral dependent current-voltage (IV) of the 350 nm T-shape (T3) p-i-n device at room temperature. **b**, Spectral dependent current at −1 V compared with expected response of InGaAs 53% adapted from literature [3].

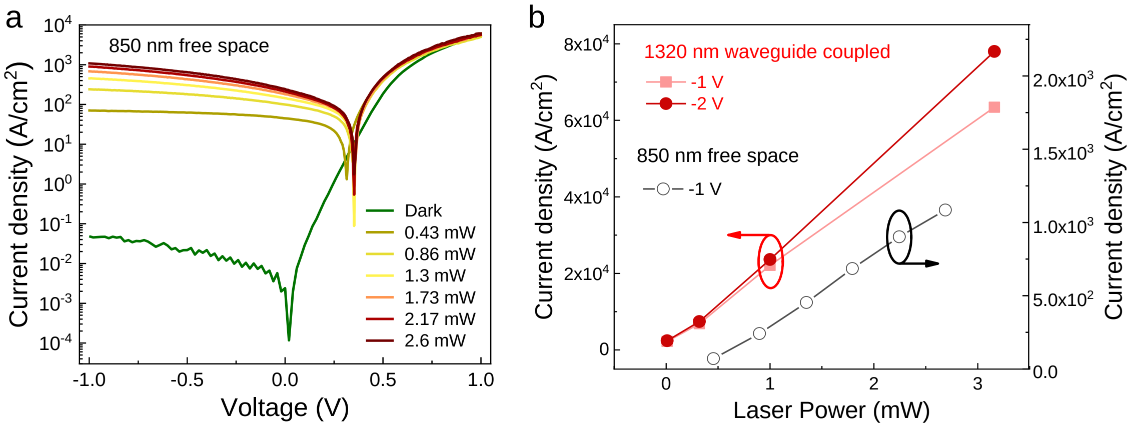

Supplementary Figure 8 Static electrical characterization of detectors. a, Free-space power dependent IV of the 350 nm T-shape (T3) p-i-n device with 850nm laser injection. **b**, Power dependent current density of wavegide coupled (1320 nm) and free-space (850 nm) laser injection.

Supplementary Figure 8a shows the power dependence of I-V curves measured in free-space with the pump laser wavelength centered at 850 nm. The current density is compared with the results measured from the waveguide coupled measurement with the laser wavelength centered

at 1320 nm in Supplementary Figure 8b. The current density increases linearly with the laser power.

Supplementary Note 4: Eletro-optical simulation

Electro-optical simulation. A 3D electromagnetic simulation is performed using CST Studio to obtain theoretical absorption efficiencies at 1320 nm. The following refractive indexes are assumed:

Supplementary Table 2 Refractive indexes for electromagnetic simulations.

Material	Real part of Refractive Index, n	Imaginary part of Refractive Index, i
Si	3.50	-
SiO_2	1.45	-
$In_{0.53}Ga_{0.47}As$	3.59	0.14
InP	3.20	-
Au	0.25	8.78
Ni	2.99	6.80

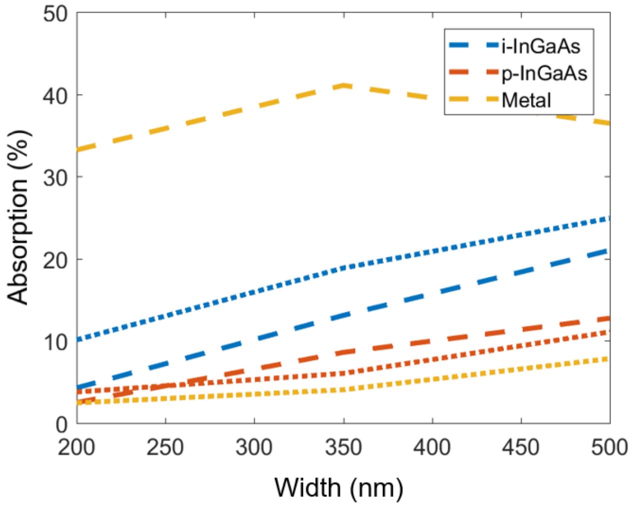

Supplementary Figure 9 Absorption in different materials. Dashed lines: straight devices, dotted lines: T-shape devices. The blue lines are for i-InGaAs, red lines are for p-InGaAs and yellow ones are for metal.

The absorption in i-InGaAs, p-InGaAs and the metal contacts are plotted in Supplementary Figure 9. All other materials have negligible absorption. For both geometries, wide devices are more efficient within the range considered here. Straight devices have higher metal losses and slightly higher absorption in the p-InGaAs. The desired absorption in the intrinsic region corresponds well with the measured responsivities.

Supplementary References

1. Menges, F., Riel, H., Stemmer, A. & Gotsmann, B. Nanoscale thermometry by scanning thermal microscopy. *Rev. Sci. Instrum.* 87, 074902 (2016).

2. Lörtscher, E., Widmer, D., Gotsmann, B. Next-generation nanotechnology laboratories with simultaneous reduction of all relevant disturbances. *Nanoscale* 5,10542-9 (2013).

3. Zimmermann, *H. Integrated Silicon Optoelectronics.* 3-5 (Springer, 2000).
